# Cabozantinib for different endocrine tumours: killing two birds with one stone. A systematic review of the literature

**DOI:** 10.1007/s12020-023-03526-0

**Published:** 2023-10-18

**Authors:** Elena Zago, Antonio Galluzzo, Silvia Pradella, Lorenzo Antonuzzo, Mario Maggi, Luisa Petrone, Clotilde Sparano

**Affiliations:** 1 Endocrinology Unit, Department of Experimental and Clinical Biomedical Sciences ‘Mario Serio’, University of Florence, Florence, Italy; 2 Department of Radiology, Careggi University Hospital, Florence, Italy; 3 Clinical Oncology Unit, Department of Experimental & Clinical Medicine, University of Florence, Florence, Italy

**Keywords:** Cabozantinib, TKI, Medullary Thyroid Carcinoma, Merkel Cell Carcinoma, Endocrine Neoplasms, Neuroendocrine Neoplasms

## Abstract

**Purpose:**

Cabozantinib is an oral multi-tyrosine kinase inhibitor (TKI) that has been approved in Europe for advanced renal cell carcinoma, hepatocellular carcinoma, locally advanced and metastatic medullary thyroid carcinoma (MTC) and radioiodine-refractory differentiated thyroid cancer. Merkel cell carcinoma (MCC) is a rare and highly aggressive cutaneous malignant neuroendocrine tumour that usually presents in sun-exposed skin areas of immunosuppressed patients. Conflicting data exist about cabozantinib for MCC and this TKI is currently under investigation in several onco-endocrine frameworks.

**Methods:**

We herein report a case of an 83-year-old man who was diagnosed with MCC during the treatment of an advanced metastatic MTC. The diagnosis of MCC was established based on clinical, histopathologic evaluation and immunohistochemistry. A systematic review of the literature on cabozantinib use for advanced endocrine and neuroendocrine tumours has been performed.

**Results:**

The patient was initially treated with surgery and adjuvant radiotherapy. Cabozantinib was therefore started to control both MTC and MCC. After 24 months, no sign of local or metastatic MCC relapse was evidenced.

**Conclusion:**

Promising data on cabozantinib treatment for endocrine and neuroendocrine neoplasms is recently emerging in the literature. In our clinical case, we reported that, besides the good response for the MTC, cabozantinib also seems to effectively control metastatic MCC, along with efficient surgery and adjuvant radiotherapy. Further investigations are needed to determine the efficacy and safety of cabozantinib in MCC patients and in off-label endocrine tumours.

## Background

**Fig. 1:**
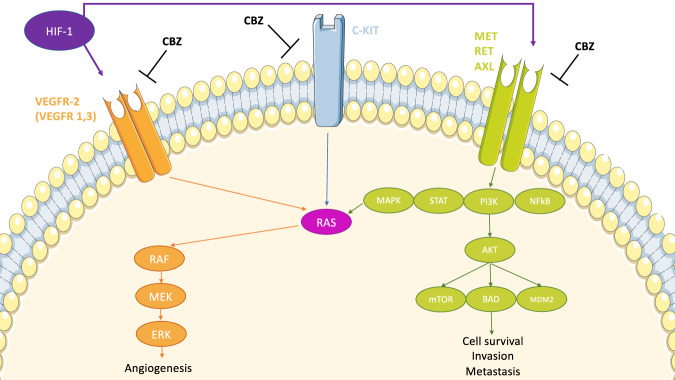
Molecular pathways inhibited by cabozantinib

The therapeutic breakthrough in the field of endocrine tumours has been the introduction of tyrosine-kinase inhibitors (TKIs) for several advanced carcinomas. Among the available drugs, cabozantinib (CBZ) has increasingly drawn attention for its wide range of benefits in progressive thyroid cancers and beyond. CBZ is an oral small molecular inhibitor of several tyrosine kinases, such as vascular endothelial growth factor receptor 2 (*VEGFR2*), tyrosine-protein kinase receptor UFO (*AXL*), mesenchymal-epidermal transition factor (*MET*), and rearranged during transfection (*RET*). Targeting intracellular key pathways is essential to hamper tumour survival, angiogenesis, epithelial-to-mesenchymal transition and other metastatic processes [1]. In particular, while *VEGFR* inhibition alone can still allow tumour spread, due to the activation of the parallel hypoxia-induced *MET* signalling, this TKI can concurrently block both pathways, resulting in enhanced disease control (Fig. 1) [1]. As regarding thyroid tumours, CBZ was firstly approved for locally advanced and metastatic medullary thyroid cancer (MTC) and later on, supported by the promising data from phase III COSMIC-311 trial, as second-line therapy for progressive radioiodine-refractory differentiated thyroid cancer (RAIR-DTC) [2, 3]. Out of on-label context, this TKI is currently under consideration for several endocrine tumours, showing interesting results. Although a few data are available, pheochromocytoma/paraganglioma (PPGL), adrenocortical carcinoma (ACC), neuroendocrine neoplasms (NENs) and Merkel cell Carcinoma (MCC) represent orphan diseases, that might benefit from this broad-based targeted therapy. Finally, CBZ represents an effective therapeutic weapon on syndromic tumours within the framework of multiple endocrine neoplasia type 2 (MEN2), a model of endocrine cancers with different primary sites, but with a common molecular signature (i.e. *RET* mutations).

To support those considerations, we hereby present a rare case of MTC and metachronous MCC, both with long disease control under CBZ.

Starting from this case report a systematic revision of current literature has been performed to summarise the available data or ongoing studies, exploring the use of CBZ in the endocrine tumours of various origins and histotypes.

## Patients and methods

The patient gave his informed consent for the present case report.

### Electronic literature search strategy

Systematic research of articles published between 2009 and 2023 on MEDLINE (PubMed), Scopus and EMBASE was performed. For search strategy and search terms refer to review protocol (Supplementary File 1). Reference lists were manually screened for further relevant articles. The search strategy was based on the PICO approach (a standardised way of defining research questions, focusing on Patients, Intervention, Comparison, and Outcome) [4]. The review was registered with PROSPERO registration number CRD42023403886 (https://www.crd.york.ac.uk/prospero/).

### Data collection and analysis

Titles and abstracts were first reviewed. Eligible studies were assessed on the basis of their full text and referenced using Zotero Software v6.0.26 (Zotero.org). Preferred Reported Items for Systematic Reviews and Meta-analyses (PRISMA) guidelines were followed (see Supplementary File 2) [5].

## Results

### Case presentation

A 79 years old man was evaluated in our tertiary referral centre in 2018 for persistently high Carcinoembryonic Antigen (CEA) levels (up to 63.3 ng/mL), observed during the follow-up for a history of colorectal adenocarcinoma (pT3pN2aM0, stage IIIB [6]), previously classified as recovered in 2017, after surgery and adjuvant chemotherapy.

An increased glucose uptake in the thyroid right lobe on 18Fluorodeoxyglucose-Positron emission tomography (FDG-PET) was revealed and neck ultrasound confirmed the presence of a 14 mm suspicious nodule on the right thyroid lobe without suspect lymph nodes in the lateral cervical districts. The following fine needle aspiration showed a TIR4/Suspicious for malignancy cytology, according to the Italian classification system for thyroid cytology [7]. Preoperative plasma calcitonin (plCT) levels resulted in 1350 pg/mL, thus suggesting the MTC diagnosis. In May 2018, the patient underwent total thyroidectomy and cervical central lymph node dissection, and the histology confirmed the diagnosis of a stage IVA MTC [pT1b (m), pN1a], according to TNM 2017 edition VIII [8]. The patient was initially classified as M0, due to the absence of distant metastases at computer tomography (CT) and FDG-PET studies. Genetic screenings for Multiple Endocrine Neoplasia 2 (MEN2) and Familial Medullary Thyroid Cancer (FMTC) were negative, but somatic *RETM918T* mutation was detected in the tumour sample.

After surgery, he didn’t reach the biochemical cure. Due to a higher risk of recurrence [9], he underwent strict radiological and biochemical monitoring and a progressive increase of plCT up to 5864 pg/mL and CEA up to 104.7 ng/mL was observed. A neck ultrasound revealed the presence of right lateral-cervical round lymph nodes suspicious for MCT locoregional metastases. Moreover, the total body CT scan showed some focal hepatic lesions in IV, VIII and VI liver segments, up to about one centimetre. A liver biopsy then confirmed the MTC origin of the metastatic lesion in November 2019.

Due to the quick progression and the multiple metastatic sites, systemic treatment was established and, despite the excellent global performance status according to Eastern Cooperative Oncology Group Performance Status (ECOG PS), considering the patient’s age, his body mass index of 25 kg/m^2^ and the clinical history, a first-line therapy with vandetanib at the personalised dose of 200 mg daily was started. The patient was systematically evaluated from biochemical, radiological and clinical perspectives, and adverse events (AE) were classified according to Common Terminology Criteria for Adverse Events (CTCAE) version 5.0 [10]. This treatment was poorly tolerated, with a grade (G) 3 asthenia and haematologic toxicity, requiring therapeutic pauses and dose reductions. However, it allowed a morphologic stabilisation of the disease, and a biochemical response with a plCT and CEA nadir of 1280 pg/mL and 61 ng/mL, respectively.

In May 2020, a new-onset right inguinal swelling was detected at the CT scan, during MTC follow-up. Owing to the uncommon tumour progression a focused histological insight was decided and the biopsy revealed a lymph node metastasis from a Merkel Cell Carcinoma (MCC). In fact, immunohistochemistry was positive for CK20, CgA, Neuron Specific Enolase (NSE) and CD56. The following investigations by FDG-PET and a new CT scan revealed a primary homolateral MCC on the right leg with locoregional lymph node metastases up to 27 mm. The biochemical assessment revealed a Chromogranin A (CgA) level of 104.9 ng/mL (range 0.0–76.3 ng/ml). Furthermore, a total body (TB) CT scan performed in September 2020, showed: stable millimetric focal hypodense hepatic lesions in IV, VIII and VI liver segments, but two new onset right lateral-cervical lymph node metastases (with anteroposterior axis of 11 and 9 mm) with contrast enhancement, and some bone rarefaction areas in the 7th and 9th thoracic vertebrae and in the 2nd lumbar vertebra, all suspicious for MTC origin; multiple external iliac and inguinal lymph nodes metastases (the largest lesions presented anteroposterior axes of 27 × 18 mm and 19 × 17 mm, respectively) of MCC origin. The patient underwent surgery on the primary MCC, and adjuvant locoregional radiotherapy (42 Gray on inguinal and external iliac lymph nodes) was performed. The MCC was finally classified as a clinical-stage III, due to the presence of *in-transit metastases* with lymph node metastases (i.e. N3 category), and accordingly, the risk of recurrence resulted as high with an estimated 5-year overall survival <30% [11]. PlCT, CEA and CgA were 6120 pg/mL, 74.1 ng/mL and 76.5 ng/mL, respectively. Due to the bad endurance and the actual diagnosis, vandetanib was definitely withdrawn.

In November 2020, considering the global oncological history with two aggressive and active endocrine tumours, second-line therapy with CBZ to control both MTC and MCC was started at the dosage of 100 mg daily but soon interrupted for a week and then titrated to 60 mg daily due to several AEs [weight loss (G2), fatigue (G1-G2), dysgeusia (G1), mild hypocalcaemia (G1), elevated blood pressure (G1), and moderate lymphopenia (G3)].

In 2021, three years after the MTC diagnosis, one year after the MCC diagnosis and three months after the switch from vandetanib to CBZ, the following TB-CT scan showed partial response disease: the previously described hepatic lesions, after an initial pseudo-progression (due to the mild swelling by drug-induced necrosis), were no longer evidenced, and the MCC iliac lymph nodes underwent a gradual downsizing, up to the complete response, according to the Response Evaluation Criteria in Solid Tumours (RECIST version 1.1) [12]. Bone metastases showed a < 20% decrease in dimension on PET-FDG and MRI. PlCT was 1010 pg/mL and CgA 137.5 ng/mL (under proton-pump inhibitor therapy).

In 2022, CBZ was again temporarily disrupted due to G4 leucopenia and G1 thrombocytopenia. Infective diseases and concomitant haematologic causes of pancytopenia were excluded. After a break of one month, the patient was in good clinical condition (ECOG PS was 0); blood pressure was controlled without medication, QTc interval was in range and the blood count was stable with only mild lymphopenia (G1), CBZ was started again at the personalised dose of 40 mg daily.

**Fig. 2:**
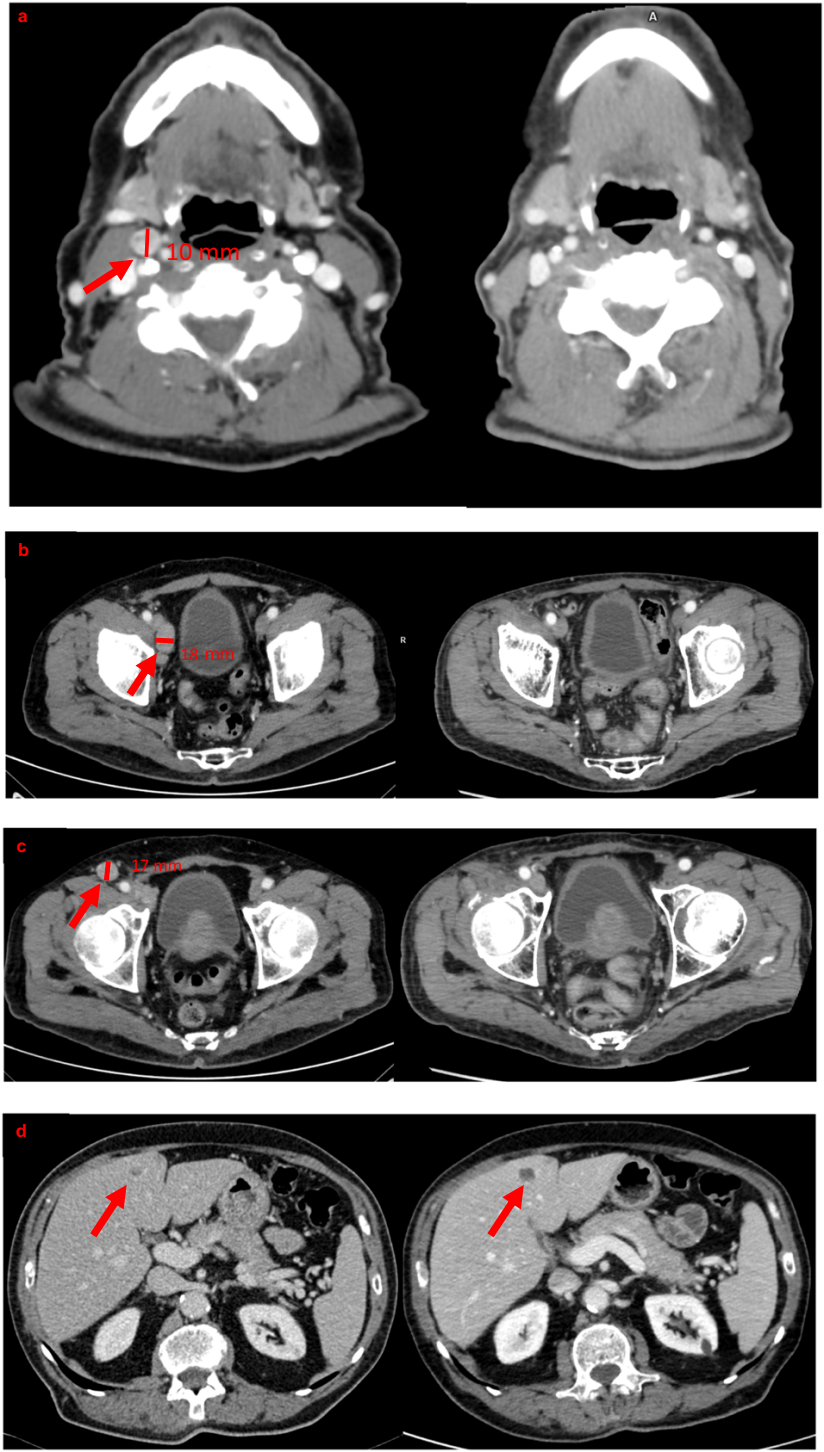
On the left baseline CT scan (before cabozantinib start); on the right CT scan of the biochemical and radiological nadir (after 22 months). **a** On the left CT scan showed pathological latero-cervical lymph node (11 × 10 mm); while on the right, no pathological lymph node is evident. **b** On the left CT scan showed right external inguinal lymph node (27 × 18 mm); while on the right, no pathological lymph node is present. **c** On the left CT scan showed right inguinal lymph node (19 × 17 mm); while on the right, no pathological lymph node is present. **d** CT scan showed pseudoprogression of hypodense hepatic lesion in the IV segment between baseline and after 22 months

**Fig. 3:**
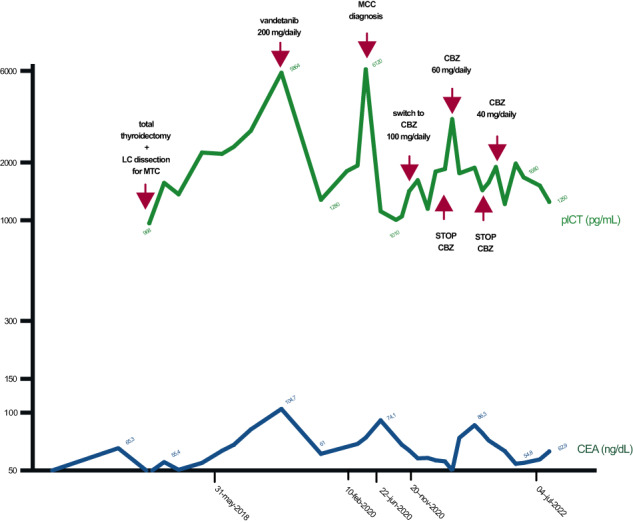
Patient’s plCT and CEA monitoring before and during treatment. LC Latero-Cervical, MCC Merkel Cell Carcinoma, CBZ cabozantinib, plCT Plasmatic Calcitonin, CEA Carcino-Embryonic Antigen

Nowadays, after four years from the MTC and two years from the MCC diagnosis, the patient, aged 83 years old, appears in good general condition (ECOG PS 0) with an excellent tolerance of this treatment, despite his age. Mild leukopenia (G1) is still present, while platelets are within the normal range. At the CT scan, MTC sustained the previous stable disease response, and no sign of local or metastatic relapses of MCC was evidenced, configuring a complete response according to RECIST criteria (Fig. 2). At the present time, plCT is 1680 pg/mL; CEA 54.8 ng/mL and CgA 275 ng/mL. Figure 3 shows the patient’s plCT and CEA monitoring before and during treatment.

### Systematic literature revision

**Fig. 4:**
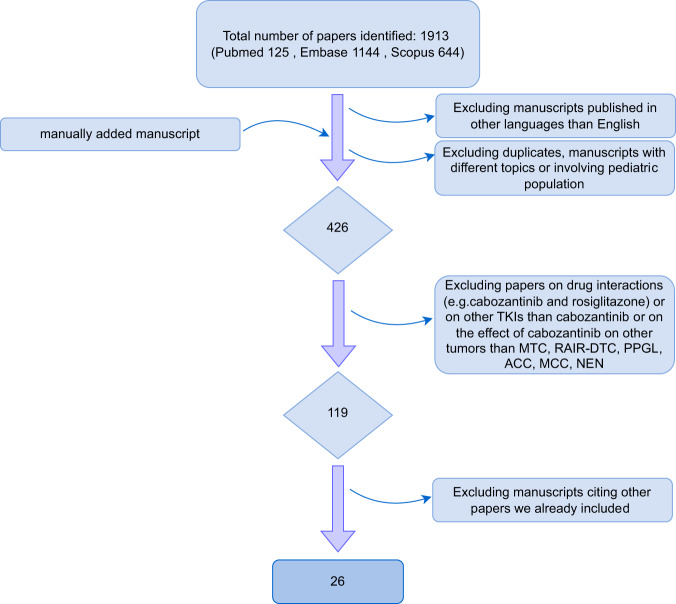
Flow diagram of the literature review process

We screened 1913 papers in the primary search. One manuscript has been manually added. After the exclusion of duplicates, studies published in other languages than English, studies involving the paediatric population and studies with different topics, 426 citations were screened for eligibility criteria on an abstract basis. 119 articles were finally analysed with a full-text review, and 26 studies were included in the analysis (Table 1). Figure 4 provides a flowchart of the literature search.

**Table 1:** List of included studies

Condition	PI	PY	Study design	Phase	Enrolled patients	Dose (mg/daily)
DTC	Cabanillas	2014	Clinical Trial	I	15	140
DTC	Shah	2015	Clinical Trial	II	25	60 or 80 (if tolerated)
DTC	Konda	2017	Prospective analysis on clinical trial	II	25	60 or 80 (if tolerated)
DTC	Brose	2021	Clinical Trial	III	187	60
DTC	Taylor	2022	Clinical Trial	Ib	102	40 or 60 (plus atezolizumab)
MTC	Elisei	2013	Clinical Trial	III	330	140
MTC	Bentzien	2013	Preclinical	preclinical	biochemical assays/mice model	N/A
MTC	Rinciog	2014	Adjusted indirect comparison	indirect comparison	N/A	N/A
MTC	Sherman	2016	Correlative analysis	indirect comparison	330	140
MTC	Schlumberger	2017	Clinical Trial	III	330	140
MTC	Koehler	2021	Real World Comparative Study	N/A	48	N/A
MTC	Capdevila	2022	Clinical Trial	IV	247	60 vs 140
MTC (+ other cancers)	Kurzrock	2011	Clinical Trial	I	37, (85)	MTD (175)
MCC	Rabinowits	2018	Clinical Trial	II	8	60 (40 or 20 if not tolerated)
MCC	Tarabadkar	2018	Case Series	N/A	5	60 reduced to 40
PPGL	Roman	2017	Review	N/A	N/A	N/A
PPGL	Jimenez	2018	Expert Opinion	N/A	N/A	N/A
PPGL	Economides	2020	Case Report	N/A	1	60/40
GU Tract Tumours	Apolo	2020	Clinical Trial	I	54	40
ACC	Phan	2015	Preclinical	N/A	biochemical assays/mice model	N/A
ACC	Kroiss	2020	Retrospective cohort study	N/A	16	60/100/140/20
ACC	Miller	2020	Retrospective cohort study	N/A	15	N/A
NEN	Sennino	2012	Preclinical	preclinical	biochemical assays/mice model	N/A
NEN	Reuther	2016	Preclinical	preclinical	biochemical assays/mice model	N/A
NEN	Chan	2018	Clinical Trial	II	35	60
NEN	Grillo	2018	Review	N/A	N/A	N/A

*PI* Principal Investigator, *PY* Publication Year, *DTC* Differentiated Thyroid Cancer, *MTC* Medullary Thyroid Carcinoma, *MCC* Merkel Cell Carcinoma, *PPGL* Pheochromocytoma and Paraganglioma, *GU* genitourinary, *ACC* Adrenocortical Carcinoma, *NEN* Neuroendocrine Neoplasm, *N/A* not applicable, *MTD* maximum tolerated dose

## Thyroid cancers

### Use of CBZ in MTC

MTC is a rare neuroendocrine disease arising from parafollicular C-cells of the thyroid, thus producing CT. It encompasses about 2–5% of all thyroid cancers, occurring as part of MEN2 syndrome in about 25% of cases and sporadically in 75% [13]. *RET* mutations can be detected at a germline level in familial forms, as well as at a somatic level in up to 50–90% of histological samples, according to literature results [14]. Irrespective of *RET* mutation status, MTC discloses poor prognosis in the event of distant metastases at diagnosis or during the follow-up [13]. At early stages, complete surgical resection represents a curative treatment; while the management of advanced and progressive MTC remains challenging [15]. Cytotoxic chemotherapy and radiotherapy showed limited efficacy, due to different mechanisms, including higher expression of Multi Drug Resistant 1 (MDR-1) proteins and enhancement of Heat Shock Protein pathways [16]. By reason of the low PDL1 expression on C-cells, disappointing results have been observed with immune-checkpoint inhibitors [17]. Besides, experience with peptide receptor radionuclide therapy (PRRT) is still limited, and an objective response is observed in only 10–15% of patients [18]. The best results in the treatment of advanced and progressive MTC came from TKI drugs. In particular, vandetanib and CBZ (Cometriq®), had been approved by the United States Food and Drug Administration (FDA) in 2011 and 2021, and by the European Medicine Agency (EMA) in 2012 and 2022, respectively. From a molecular perspective, in vitro and in vivo studies on mouse models demonstrated that, besides the robust *MET* and *VEGFR2* antagonism, CBZ inhibits both the wild type and the mutated forms of *RET*, preventing MTC cells and xenograft from growing and metastasising [19]. As regards clinical trials, in phase I (Study of XL184 (Cabozantinib) in Adults With Advanced Malignancies) and III trials (EXAM trial), CBZ showed an acceptable safety profile (Table 2), a longer progression-free survival (PFS) (11.2 months versus 4.0 months in the placebo arm, *p* < 0.001) and overall survival (OS) (26.6 versus 21.1 months, although the difference did not reach statistical significance, *p* = 0.24) [20, 21]. These promising results were obtained mainly in patients with *RETM918T* mutation (which was present in almost 65–70% of the tumours) [22]. In fact, according to the exploratory analysis of this study, *RETM918T* subgroup achieved the greatest observed PFS benefit for CBZ versus placebo (HR, 0.15; 95% CI, 0.08–0.28; *P* < 0.0001) [23]. Furthermore, an indirect treatment comparison between CBZ and vandetanib showed a positive trend in favour of CBZ in terms of PFS [24]. As regarding adverse effects, CBZ showed a dose-dependent relation but the EXAMINER trial failed to prove the non-inferiority of the 60 mg/day tablets versus the 140 mg/day capsules [25]. Moreover, Koehler et al. study found that both PFS and OS were significantly longer in younger patients experiencing more than five AEs, thus suggesting a dose-related correlation with the tumour response [26]. In conclusion, in MTC, CBZ appeared to be effective in prolonging survival, although many patients often do not receive full dosage or need a discontinuation of the treatment due to poor tolerance or toxicities (Table 2).

**Table 2:** Cabozantinib related adverse events [21, 34, 83]

Frequent AEs (>50%)	All grades (%)	G3 (%)	Common AEs (50-10%)	All grades (%)	G3 (%)	Not common AEs (<10%)	All grades (%)	G3 (%)
*Cardiovascular*								
hypertension	33–53	8.4–20				pulmonary embolism	3.3	0
*Dermatological*								
palmar-plantar erythrodyesthesia	40–53	13–12	rash	19.2–23	0.9			
mucositis	20–53	0–3.3	dry skin	19.2–20.1	0–2.8			
*Gastrointestinal*								
diarrhoea	52–87	7.3–21	dysgeusia	26–45	0.1–0.5			
decreased appetite	49–80	7–13	constipation	26–34	0-1			
nausea	43–80	0–6.9	stomatitis	19–30	1.4–2.6			
vomiting	24–60	2.3–7						
*Constitutional*								
fatigue	41–80	7–13	arthralgia	14	2.3			
weight decrease	35–73	3.4–13	myalgia	7–27	1			
			insomnia	10.7	0			
			pruritus	13.6	0.5			
			headache	13–18.2	0.4–0.5			
			alopecia	16.4	0			
			hair colour changes	33–34	0.5			
*Renal*								
			proteinuria	47	7			
*Lab alteration*								
incresed AST	10–100	7–3.2	hypophosphatemia	20	13	increased ALP	3	0
increased ALT	24–73	7-5	hyponatremia	13–33	7–27			
increased LDH	73	7						
hypocalcemia	23–60	13-0						
hypokalemia	47	13-3.8						
*Haematologic*								
			anaemia	20–31	7			
			leukopenia	27	7			
*Respiratory tract*								
			pneumonia	4.2–13	13			
			dyspnoea	13.6–21	2.3–3.5			
			dysphonia	21–27	0–10.1			
*Thyroid*								
			thyroid disfunction	14	0			

*AE* Adverse Event, *AST* Aspartate Aminotransferase, *ALT* Alanine Aminotransferase, *LDH* Lactate Dehydrogenase, *ALP* Alkaline Phosphatase

### Use of CBZ in RAIR-DTC

DTCs, which account for about 90% of all thyroid cancers, usually have favourable long-term prognosis following surgery±radioiodine treatment, with mortality rates ranging from 0.3–0.5/100,000 population [27, 28].

However, two-thirds of patients with recurrent or metastatic DTC are RAIR with a 10-year survival rate of only 10% after diagnosis, resulting in a challenge for physicians [29].

While indolent and oligometastatic RAIR-DTC can be followed up with suppressive levothyroxine dose alone; symptomatic, progressive low-burden disease may benefit from locoregional therapies i.e., surgery, external radiotherapy, or thermal ablation. The major challenge lies in rapidly progressive and inoperable cases or high-burden metastatic RAIR-DTC, which requires systemic therapies. In those patients, the treatment with cytotoxic chemotherapy showed disappointing results, with selective benefits only in patients who fail to respond to TKIs (data are mainly anecdotal) [30]. Hence, TKIs are considered in the event of disease progression, mainly after surgical and radiation therapy approaches [31]. Until fairly recently, lenvatinib, a wide spectrum anti-angiogenetic TKI, was the only available option for advanced RAIR-DTC and, at the time of progression to this drug, the therapeutic chances were poor [32]. In 2021 and 2022, CBZ (Cabometyx®) was finally approved by FDA and EMA for RAIR-DTC patients, who disclosed progressive disease after prior treatments with *VEGFR* inhibitors. This endorsement was based on the promising data from the COSMIC-311 study: a multicentral, randomised, double-blind trial in which patients, with locally advanced or metastatic RAIR-DTC, progressing during or after treatment with at least one *VEGFR*-targeting TKI, were treated with either CBZ 60 mg orally once daily or placebo [3]. The median PFS was 11.0 months [95% confidence interval (CI), 7.4–13.8] in the CBZ arm compared with 1.9 months (95% CI, 1.9–3.7) in the control arm [3]. The final analysis of COSMIC-311 with longer follow-up confirmed the superiority of CBZ versus placebo with a manageable safety profile [3]. The PFS benefit was consistent with the interim analysis and irrespective of prior *VEGFR*-targeted therapy [33]. Phase I and II trials that preceded the COSMIC trial showed that CBZ presented a safety profile similar to other multitargeted *VEGFR* and clinical and statistically significant activity in DTC patients who have progressed on prior *VEGFR* targeted therapies [34–36]. Furthermore, Konda et al. performed a prospective analysis as part of a multicenter International Thyroid Oncology Group phase II study of CBZ in patients with RAIR-DTC with bone metastases who progressed on prior *VEGFR*‐targeted therapy: CBZ was associated with a highly significant reduction in all bone turnover markers (even when they were within normal limits at baseline) and a minor decreases in bone lesion uptake was noted on Sodium Fluoride PET in four out of six patients [37]. Finally, CBZ provided the same efficacy advantage when in monotherapy and when associated with immune-checkpoint inhibitors such as nivolumab and ipilimumab, while it showed a durable response and high rate of disease control when in combination with atezolizumab [38, 39]. In 2018, Brose et al. presented the first study documenting CBZ anti-tumoral activity in patients with RAIR-DTC as a first-line therapy [40]. A phase I trial on the simultaneous use of CBZ and PD-1 inhibitors in HIV-positive patients is now recruiting (Table 3 - https://clinicaltrials.gov).

**Table 3:** List of active trials

Condition	Identifier	Status	Study Start Date	Study Completion Date	Ph	EP	Primary Objectives	Drugs
RAIR- DTC	NCT05660954	not yet recruiting	April, 2023	June, 2025	II	41	Gene expression	cabozantinib
DTC	NCT03914300	active, not recruiting	July 15, 2019	January 15, 2024	II	27	ORR	cabozantinib, nivolumab and ipilimumab
RAIR- DTC	NCT02041260	active, not recruiting	January 2014	May 2022	II	43	AEs	cabozantinib
Advanced DTC (+others)	NCT04514484	recruiting	November 4, 2020	November 2, 2025	I	18	DLTs	cabozantinib and nivolumab
DTC (+others)	NCT03170960	active, not recruiting	September 5, 2017	August 2024	Ib	1732	MTD	cabozantinib and atezolizumab
PPGL	NCT02302833	recruiting	February 17, 2015	December 31, 2023	II	22	ORR	cabozantinib
ACC	NCT03612232	recruiting	June 4, 2019	April 30, 2025	II	37	PFS	cabozantinib
ACC	NCT03370718	active, not recruiting	February 26, 2018	February 28, 2026	II	18	PFS	cabozantinib
NEN	NCT03375320	recruiting	July 18, 2018	October 1, 2025	III	395	PFS	cabozantinib
NEC	NCT04079712	active, not recruiting	January 14, 2020	October 1, 2023	II	30	ORR	cabozantinib, nivolumab, and ipilimumab
NEN	NCT04412629	recruiting	November 24, 2020	July 31, 2025	II	32	ORR	cabozantinib
NEN	NCT04893785	recruiting	June 15, 2021	December 2024	II	35	ORR	cabozantinib, temozolomide
NEN	NCT04427787	recruiting	June 20, 2020	November 2023	II	69	ORR	cabozantinib, lanreotide
NEN	NCT05249114	recruiting	December 28, 2022	December 1, 2031	Ib	90	MTD	cabozantinib, PRRT
Carcinoid Tumours	NCT04197310	active, not recruiting	December 26, 2019	December 26, 2023	II	35	ORR	cabozantinib, nivolumab
NEN, PPGL, ATC, Adenocarcinoma	NCT04400474	active, not recruiting	October 7, 2020	March 2024	II	93	ORR	cabozantinib, atezolizumab
NEN	NCT05048901	recruiting	September 17, 2021	December 31, 2025	I/II	49	MTD, PFS	cabozantinib
NEN G3	NCT04524208	recruiting	March 1, 2021	June 30, 2023	II	40	DCR	cabozantinib
NEN G3	NCT05289856	recruiting	March 28, 2022	December 1, 2025	II	30	DCR	cabozantinib, avelumab

*RAIR* Radioiodine-Refractory, *DTC* Differentiated Thyroid Carcinoma, *PPGL* Pheochromocytoma and Paraganglioma, *ACC* Adrenocortical Carcinoma, *NEN* Neuroendocrine Neoplasm, *ATC* Anaplastic Thyroid Carcinoma, *NEC* Neuroendocrine Carcinoma, *Ph* Phase, *ER* Enrolled Patients, *DLTs* Dose Limiting Toxicities, *ORR* Overall Response Rate, *PFS* Progression Free Survival, *MTD* Maximum Tolerated Dose, *DCT* Disease Control Rate, *G* grade

### Use of CBZ in MCC

MCC is a rare, highly aggressive neuroendocrine skin tumour that usually develops in sun-exposed areas [41]. Despite major advantages in understanding its carcinogenesis process, the MCC origin has not been completely understood: on one hand, Merkel cell precursor has been speculated as a source of MCC (normal Merkel Cells are terminally differentiated, thus allegedly not able to devolve into cancer); on the other hand, pro-B/pre-B lymphocytes, fibroblasts, dermal mesenchymal stem cells or aberrant keratinocytes have also been suspected as oncological precursors [42].

Elderly subjects with a story of sun exposition and/or immunosuppressant therapy are at higher risk for this disease [43, 44]. Local and regional lymph node metastasis can occur at the early stages, and the long-term prognosis is poor, dropping to 14% at five years from the diagnosis in the event of metastatic disease [11, 45]. Whether possible, first-line therapy encompasses a wide tumour excision, along with sentinel lymph node dissection and/or lymphadenectomy. High-dose adjuvant radiotherapy (50–60 Gray) to the primary site is usually delivered during the post-operative takeover, but this treatment also seems beneficial for locoregional control in patients with unresectable tumours [45, 46]. The historically favoured systemic therapy consisted of carboplatin/cisplatin-etoposide chemotherapy, while any second lines were based on anthracyclines, cyclophosphamide, vincristine, bleomycin, and 5-fluorouracil regimens [47]. Although cytotoxic chemotherapy has an objective response rate of >50%, responses are rarely durable with a median PFS of only three months [44]. Moreover, several safety concerns are well-known, making the traditional regimens mostly reserved for palliative care, i.e. patients with advanced or locally recurrent MCC [48]. During the last few years, thanks to the advancement in immunotherapy, different molecules have been proposed: immune-checkpoint inhibitors (PD-1 and PDL-1 ligand inhibitors) are the favoured agents. In particular, first-line therapy with avelumab showed a PFS in up to 72% of the cases in an international multicentre phase II trial (JAVELIN Merkel 200) [49]. Despite the success of immune therapy, a considerable proportion of patients with metastatic MCC do not respond to PD-1/PD-L1 blockade [49]. In this setting, TKIs have been proposed due to their capability of targeting angiogenesis and tumour-spreading processes as MCC expresses *VEGF-A*, *VEGF-C*, *VEGF-R2*, and platelet-derived growth factor (*PDGF*)-b in 91, 75, 88 and 72%, respectively [50, 51]. Despite these promising data, very little literature regarding anti-*VEGF* therapy on MCC is available: Tarabadkar described five cases of successful use of anti-*VEGF* TKIs such as pazopanib and CBZ in patients with advanced MCC [52]. In particular, a single case report of a 3.5-year PFS in a patient receiving CBZ after platinum-based chemotherapy has been described. Nonetheless, Rabinowits et al. failed to demonstrate the efficacy of single-agent CBZ and the 8 patients-trial prematurely failed, because of the lack of efficacy and toxicity [53]. However, all the included patients have been already pre-treated with heavy chemotherapy regimens - such as platin-etoposide - and received CBZ as a second or further line, which could have affected the safety and the tolerance to this drug. After all, no trial on CBZ in MCC is currently ongoing, since the phase II study on CBZ in recurrent MCC (NCT02036476), failed for lack of recruital.

### Use of CBZ in PPGLs

Pheochromocytomas and paragangliomas (PPGLs) represent a rare group of neoplasia issued from chromaffin cells. In particular, pheochromocytomas arise from the adrenal medulla, while extra-adrenal lesions develop from the extra-adrenal paraganglia.

Surgical removal is the therapy of choice in PPGLs with either a curative intent in non-metastatic disease or, in metastatic disease, to minimise symptoms derived from catecholamine excess and from compression of the surrounding structures [54]. Surgical-related morbidity, however, needs to be carefully evaluated in particular in head and neck non-functioning PPGL. In fact, the closeness to cranial nerves and vases often induces to favour a non-surgical approach, such as watchful waiting or radiosurgery (gamma-knife/cyberknife), in the event of compression of the surrounding structures [55]. Aside from radiotherapeutic options, the only officially approved treatment for advanced tumours is the high-specific iodine-131-meta-iodobenzylguanidine [(131 I)MIBG] therapy (Ultratrace), while chemotherapy and different targeted therapies are widely used outside controlled clinical trials [56–58]. Recently, TKIs have been in the spotlight, due to their ability to inhibit tumour neoangiogenesis. Furthermore, they also seemed to play a role in the bone microenvironment, by interfering with bone turnover, thus reducing eventual pain [59].

So far, the best studied TKI is sunitinib, which seems to have better efficacy in *RET* and *SDHx* mutated patients: in a large prospective phase II multicentre study, the total disease control rate (including stable disease or partial response) in patients with germline mutations (*SDHA, SDHB, RET*) was 83% (95% CI: 61–95%) [60]. At the moment, sunitinib is under investigation in the first randomised placebo-controlled phase II clinical trial in advanced PPGLs (FIRSTMAPPP, NCT01371202). Despite the promising data on sunitinib, other TKI such as CBZ have not yet been completely studied. In fact, in the literature, there is a case report of a 32-year-old male patient suffering from an advanced metastatic paraganglioma, showing a progressive disease when enrolled in a CBZ-based trial as a monotherapy treatment. Nonetheless, he showed prolonged disease control with a significant tumour reduction and decrease in metastatic burden when enrolled in the study by Apolo (started on CBZ plus nivolumab) [61, 62]. A phase II clinical trial on CBZ in monotherapy is now recruiting patients with unresectable metastatic paraganglioma or pheochromocytoma (Table 3 - https://clinicaltrials.gov). Preliminary results on patients treated with 60 mg daily (titrated down to 40 or 20 mg depending on the tolerability) showed tumour mass shrinkage and stability of the disease [63]. Moreover, a phase II trial on the combination therapy (CBZ plus atezolizumab) in patients suffering from advanced and progressive neoplasms of the endocrine system is ongoing, although not actively recruiting (Table 3 - https://clinicaltrials.gov).

### Use of CBZ in ACC

Adrenocortical carcinoma (ACC) is a rare tumour arising from the adrenocortical gland, usually producing steroid hormones [64]. The prognosis is often poor since patients at low risk of recurrence are the minority and they can also frequently further develop recurrent disease [65]. In localised stages, complete surgical resection is the treatment of choice, while mitotane is the only approved drug that showed efficacy in prolonging recurrence-free survival when administrated as adjuvant treatment in patients at higher risk of recurrence [66]. Other adjuvant therapies, including chemotherapy, had been under investigation: although the European Society of Endocrinology did not reach a definitive consensus on adjuvant use of cytotoxic drugs for ACC, based on phase III trial, the combination of etoposide, doxorubicin and cisplatin (EDP) with mitotane (EDP-M) is recommended in patients with very high risk of recurrence [67]. Although the rates of response and PFS were significantly better with EDP-M treated patients, the study failed to show a significant difference in terms of overall survival [67]. In this setting, TKIs have been considered as a second-line therapy for patients who showed disease progression under EDP-M [68]. The rationale for the use of TKIs is based on the study of Pnan et al. who demonstrated an increased phosphorylation of *c-MET* in the immunohistochemistry of ACC tissue samples compared to adrenocortical adenomas and normal cortex, thus speculating that targeting *c-MET* could provide a breakthrough in the management of these aggressive diseases [69]. Moreover, an in vivo experiment in a mouse model confirmed that the knockout of *c-MET* inhibited adrenocortical carcinoma growth, tumour-related angiogenesis, chemotherapy resistance and cell survival, further suggesting this molecular pathway as a valuable therapeutic target for adrenocortical carcinoma [69]. Based on those promising preclinical data, a retrospective cohort study on CBZ use in patients with progressive disease under mitotane or cytotoxic chemotherapy regimens has been performed: best response was partial response in three out of 16 subjects, stable disease in five and progressive disease in eight patients. Although eight subjects (50% of the patients) showed tumour progression, this result was considered favourable (PFS was 16 weeks and OS was 58 weeks), compared to other treatments that failed to demonstrate a prolonged overall survival [68, 70]. A single institution experience showed a stable disease lasting six months in one patient treated with CBZ after previously progressing through chemotherapy and pembrolizumab [71].

To date, there is a phase II active trial actively recruiting patients suffering from unresectable or metastatic ACC in order to assess the efficacy and safety of CBZ (Table 3*-* www.ClinicalTrials.gov).

### Use of CBZ in NENs

Neuroendocrine neoplasms (NENs) are a heterogeneous group of neoplasms originating from neuroendocrine cells localised in different organs, mostly within the digestive tract and in the lungs [72]. Therapeutic options rely on the tumour’s primary site, morphology, grade and stage, encompassing the “watchful and wait” attitude, surgical resection, locoregional treatment, or systemic therapies, including somatostatin receptor analogues, targeted therapies or traditional chemotherapy, in the event of advanced and progressive disease not responding to other treatments [73–75]. As regards multi-TKIs, sunitinib is the only one that obtained EMA and FDA approval for pancreatic NENs, while other TKIs are currently under evaluation [76]. In particular, Reuther and colleagues found out that, while *c-MET* inhibition alone is not sufficient to exert direct antitumor or antimigratory effects in NEN cells, the multi-TKIs CBZ, as well as tivantinib, disclose therapeutic efficacy, probably due to the suppression of other molecular pathways [77]. Preclinical studies showed a reduced invasion and metastatic activity in mice with pancreatic NEN treated with CBZ: in particular, the simultaneous inhibition of *VEGFR* and *MET* pathways resulted in a smaller tumour mass and no metastases for CBZ-treated mice, compared to only anti-*VEGF*-treated mice [78]. The multi-TKI CBZ has also shown promising antitumor activity in a preliminary report of a phase II trial for pancreatic and extra-pancreatic NENs, showing a PFS of 21.8 months and 31.4 months in patients with pancreatic and non-pancreatic NENs, respectively [79]. Based on those results, five clinical trials are actively recruiting while three more trials are still active but not recruiting (Table 3 - www.ClinicalTrials.gov) [80].

## Discussion

To the best of our knowledge, very few data on CBZ in endocrine and neuroendocrine tumours are available so far, while it is widely agreed that TKI improved MTC and differentiated thyroid cancer’s natural history. In the present case report, CBZ, along with a multimodal therapeutic approach, including surgery and adjuvant radiotherapy, showed a 24-month PFS from both MTC and MCC in an 83-year-old man. Of note, this patient disclosed two highly aggressive endocrine tumours, i.e. IV-stage MTC and a high-recurrence risk MCC with an estimated 5-year survival of <30%. For these reasons, it was necessary to provide a broad-spectrum treatment, able to control both these diseases. CBZ appeared as the better compromise, even if its efficacy on MCC is still uncertain. In fact, literature data are scant and contrasting, but it is worth noting that the failed focused trial on CBZ for MCC [53] was significantly biased by the heavy systemic chemotherapy treatments performed by the included patients, at odds with the present patient’s history.

Although globally safe and well-tolerated, CBZ-related adverse events often need a tailored approach with personalized doses and frequent clinical, biochemical and radiological follow-ups. In the present experience, CBZ, as a second-line therapy, was interrupted for some days or weeks when the patient showed lower tolerance, and the dose was also adjusted up to 40 mg daily without affecting the PFS.

Different neoplasms with higher metastatic load are effectively controlled under targeted therapies and many endocrine tumours include multi-targeted or selective TKI in their protocols. These treatments can produce different adverse events, i.e. variable rates of myelosuppression and immunodepression, along with anorexia, fatigue, nausea, anomalies in liver function test, hypertension, thrombocytopenia, palmar-plantar erythrodysesthesia, QTc prolongation, notably in elderly subjects [22]. As a matter of fact, despite the high prevalence of cancer in elderly people, very few data on antineoplastic therapy effects in this specific population are available [81]. Clinical trials, in fact, usually include a very selected population and, as a consequence, the experience with targeted therapy is limited in the geriatric population. On the other hand, it is well-known that about 30–50% of elderly patients will experience severe side effects of antineoplastic therapy, which is further increased when combined regimens are used [82].

Rare endocrine diseases are often orphan diseases but with overlapping effectiveness of some drugs. It is probably the case of CBZ, which has been tested with benefits in the thyroid cancer field of both follicular and neuroendocrine origins. Moreover, a somewhat therapeutic potential also emerged in other advanced endocrine tumours, although the conclusion on its effectiveness is often biased by the presence of former heavy treatment regimes. However, MCC, adrenal, chromaffin and neuroendocrine tumours have been or currently are under investigation for this TKI. The endocrine tumour framework is wide and complex, however, several touchpoints in the molecular pathways and therapies emerged from preclinical models to real practice. We are aware that streamlining the therapeutic approach in this various disease spectrum is probably not fully viable, since each tumour retains its peculiarities and uniqueness. However, to speed up the insight into new drugs’ effectiveness on different and rare endocrine tumours, a new clinical trial concept is desired, switching between a tumour-specific study mindset to a comprehensive and wide-inclusive endocrine tumour model.

### Supplementary information

Supplementary File 1

Supplementary File 2

## References

[1] Yakes FM, Chen J, Tan J, Yamaguchi K, Shi Y, Yu P, Qian F, Chu F, Bentzien F, Cancilla B, Orf J, You A, Laird AD, Engst S, Lee L, Lesch J, Chou Y-C, Joly AH (2011). Cabozantinib (XL184), a Novel MET and VEGFR2 Inhibitor, Simultaneously Suppresses Metastasis, Angiogenesis, and Tumor Growth. Mol. Cancer Ther..

[2] Fallahi P, Ferrari S, Bari F, Materazzi G, Benvenga S, Miccoli P, Antonelli A (2015). Cabozantinib in Thyroid Cancer. Recent Pat. Anticancer Drug Discov..

[3] Brose MS, Robinson B, Sherman SI, Krajewska J, Lin C-C, Vaisman F, Hoff AO, Hitre E, Bowles DW, Hernando J, Faoro L, Banerjee K, Oliver JW, Keam B, Capdevila J (2021). Cabozantinib for radioiodine-refractory differentiated thyroid cancer (COSMIC-311): a randomised, double-blind, placebo-controlled, phase 3 trial. Lancet Oncol..

[4] Schardt C, Adams MB, Owens T, Keitz S, Fontelo P (2007). Utilization of the PICO framework to improve searching PubMed for clinical questions. BMC Med. Inform. Decis. Mak..

[5] Liberati A, Altman DG, Tetzlaff J, Mulrow C, Gotzsche PC, Ioannidis JPA, Clarke M, Devereaux PJ, Kleijnen J, Moher D (2009). The PRISMA statement for reporting systematic reviews and meta-analyses of studies that evaluate healthcare interventions: explanation and elaboration. BMJ.

[6] Amin MB, Greene FL, Edge SB, Compton CC, Gershenwald JE, Brookland RK, Meyer L, Gress DM, Byrd DR, Winchester DP (2017). The Eighth Edition AJCC Cancer Staging Manual: Continuing to build a bridge from a popula-based to a more “personalized” approach to cancer staging: The Eighth Edition AJCC Cancer Staging Manual, CA. Cancer J. Clin..

[7] Nardi F, Basolo F, Crescenzi A (2014). Italian consensus for the classification and reporting of thyroid cytology. J. Endocrinol. Investig..

[8] M. Tuttle, L.F. Morris, B. Haugen, J. Shah, J.A. Sosa, E. Rohren, R.M. Subramaniam, J.L. Hunt, N.D. Perrier, M.B. Amin, S.B. Edge, F. Greene, D. Byrd, R.K. Brookland, M.K. Washington, C.C. Compton, K.R. Hess, D.C. Sullivan, J.M. Jessup, Thyroid-differentiated and anaplastic carcinoma (Chapter 73), Springer International Publishing, n.dA.

[9] Sparano C, Adornato V, Puccioni M, Zago E, Perigli G, Badii B, Santoro R, Maggi M, Petrone L (2023). Early calcitonin levels in medullary thyroid carcinoma: Prognostic role in patients without distant metastases at diagnosis. Front. Oncol..

[10] Common Terminology Criteria for Adverse Events (CTCAE), (2017)

[11] Gauci M-L, Aristei C, Becker JC, Blom A, Bataille V, Dreno B, Del Marmol V, Forsea AM, Fargnoli MC, Grob J-J, Gomes F, Hauschild A, Hoeller C, Harwood C, Kelleners-Smeets N, Kaufmann R, Lallas A, Malvehy J, Moreno-Ramirez D, Peris K, Pellacani G, Saiag P, Stratigos AJ, Vieira R, Zalaudek I, Van Akkooi ACJ, Lorigan P, Garbe C, Lebbé C (2022). Diagnosis and treatment of Merkel cell carcinoma: European consensus-based interdisciplinary guideline – Update 2022. Eur. J. Cancer.

[12] Eisenhauer EA, Therasse P, Bogaerts J, Schwartz LH, Sargent D, Ford R, Dancey J, Arbuck S, Gwyther S, Mooney M, Rubinstein L, Shankar L, Dodd L, Kaplan R, Lacombe D, Verweij J (2009). New response evaluation criteria in solid tumours: Revised RECIST guideline (version 1.1). Eur. J. Cancer.

[13] Wells SA, Asa SL, Dralle H, Elisei R, Evans DB, Gagel RF, Lee N, Machens A, Moley JF, Pacini F, Raue F, Frank-Raue K, Robinson B, Rosenthal MS, Santoro M, Schlumberger M, Shah M, Waguespack SG (2015). Revised American Thyroid Association Guidelines for the Management of Medullary Thyroid Carcinoma: The American Thyroid Association Guidelines Task Force on Medullary Thyroid Carcinoma. Thyroid.

[14] Lodish MB, Stratakis CA (2008). RET oncogene in MEN2, MEN2B, MTC and other forms of thyroid cancer. Expert Rev. Anticancer Ther..

[15] L. Fugazzola, Medullary thyroid cancer - An update, Best Pract. Res. Clin. Endocrinol. Metab. 37 (2023). 10.1016/j.beem.2022.101655. 10.1016/j.beem.2022.101655 35422397

[16] Ruggeri RM, Sciacchitano S, Vitarelli E, Trimarchi F, Barresi G, Trovato M (2006). Immunoexpression of Multidrug-Resistance Protein 2 and Cyclooxygenase 2 in Medullary Thyroid Carcinomas. Arch. Pathol. Lab. Med..

[17] A. Hegde, A.Y. Andreev-Drakhlin, J. Roszik, L. Huang, S. Liu, K. Hess, M. Cabanillas, M.I. Hu, N.L. Busaidy, S.I. Sherman, R. Dadu, E.G. Grubbs, S.M. Ali, J. Lee, Y.Y. Elamin, G.R. Simon, G.R. Blumenschein, V.A. Papadimitrakopoulou, D. Hong, F. Meric-Bernstam, J. Heymach, V. Subbiah, Responsiveness to immune checkpoint inhibitors versus other systemic therapies in RET-aberrant malignancies, ESMO Open. 5 (2020). 10.1136/esmoopen-2020-000799. 10.1136/esmoopen-2020-000799 PMC7590373 33097651

[18] Maghsoomi Z, Emami Z, Malboosbaf R, Malek M, Khamseh ME (2021). Efficacy and safety of peptide receptor radionuclide therapy in advanced radioiodine-refractory differentiated thyroid cancer and metastatic medullary thyroid cancer: a systematic review. BMC Cancer.

[19] Bentzien F, Zuzow M, Heald N, Gibson A, Shi Y, Goon L, Yu P, Engst S, Zhang W, Huang D, Zhao L, Vysotskaia V, Chu F, Bautista R, Cancilla B, Lamb P, Joly AH, Yakes FM (2013). In vitro and in vivo activity of cabozantinib (XL184), an inhibitor of RET, MET, and VEGFR2, in a model of medullary thyroid cancer. Thyroid.

[20] Kurzrock R, Sherman SI, Ball DW, Forastiere AA, Cohen RB, Mehra R, Pfister DG, Cohen EEW, Janisch L, Nauling F, Hong DS, Ng CS, Ye L, Gagel RF, Frye J, Müller T, Ratain MJ, Salgia R (2011). Activity of XL184 (Cabozantinib), an Oral Tyrosine Kinase Inhibitor, in Patients With Medullary Thyroid Cancer. J. Clin. Oncol..

[21] Schlumberger M, Elisei R, Müller S, Schöffski P, Brose M, Shah M, Licitra L, Krajewska J, Kreissl MC, Niederle B, Cohen EEW, Wirth L, Ali H, Clary DO, Yaron Y, Mangeshkar M, Ball D, Nelkin B, Sherman S (2017). Overall survival analysis of EXAM, a phase III trial of cabozantinib in patients with radiographically progressive medullary thyroid carcinoma. Ann. Oncol..

[22] Elisei R, Schlumberger MJ, Müller SP, Schöffski P, Brose MS, Shah MH, Licitra L, Jarzab B, Medvedev V, Kreissl MC, Niederle B, Cohen EEW, Wirth LJ, Ali H, Hessel C, Yaron Y, Ball D, Nelkin B, Sherman SI (2013). Cabozantinib in Progressive Medullary Thyroid Cancer. J. Clin. Oncol..

[23] Sherman SI, Clary DO, Elisei R, Schlumberger MJ, Cohen EEW, Schöffski P, Wirth LJ, Mangeshkar M, Aftab DT, Brose MS (2016). Correlative analyses of RET and RAS mutations in a phase 3 trial of cabozantinib in patients with progressive, metastatic medullary thyroid cancer. Cancer.

[24] Rinciog C, Myrén KJ, Aldén M, Diamantopoulos A, Le Reun C (2014). An indirect treatment comparison of cabozantinib verse vandetan ib in progressive medullary thyroid cancer (MTC). Value Health.

[25] Capdevila J, Klochikhin A, Leboulleux S, Isaev P, Badiu C, Robinson B, Hughes BGM, Keam B, Parnis F, Elisei R, Gajate P, Gan HK, Kapiteijn E, Locati L, Mangeshkar M, Faoro L, Krajewska J, Jarzab B (2022). A Randomized, Double-Blind Noninferiority Study to Evaluate the Efficacy of the Cabozantinib Tablet at 60 mg per Day Compared with the Cabozantinib Capsule at 140 mg per Day in Patients with Progressive, Metastatic Medullary Thyroid Cancer. Thyroid.

[26] Koehler VF, Adam P, Frank-Raue K, Raue F, Berg E, Hoster E, Allelein S, Schott M, Kroiss M, Spitzweg C, on Behalf of the German Study Group for Rare Malignant Tumors of the Thyroid and Parathyroid Glands (2021). Real-World Efficacy and Safety of Cabozantinib and Vandetanib in Advanced Medullary Thyroid Cancer. Thyroid.

[27] Sparano C, Moog S, Hadoux J, Dupuy C, Al Ghuzlan A, Breuskin I, Guerlain J, Hartl D, Baudin E, Lamartina L (2022). Strategies for Radioiodine Treatment: What’s New. Cancers.

[28] Sung H, Ferlay J, Siegel RL, Laversanne M, Soerjomataram I, Jemal A, Bray F (2021). Global Cancer Statistics 2020: GLOBOCAN Estimates of Incidence and Mortality Worldwide for 36 Cancers in 185 Countries, CA. Cancer J. Clin..

[29] Schmidt A, Iglesias L, Klain M, Pitoia F, Schlumberger MJ (2017). Radioactive iodine-refractory differentiated thyroid cancer: an uncommon but challenging situation. Arch. Endocrinol. Metab..

[30] Droz J-P, Schlumberger M, Rougier P, Ghosn M, Gardet P, Parmentier C (1990). Chemotherapy in Metastatic Nonanaplastic Thyroid Cancer: Experience at the Institut Gustave-Roussy. Tumor. J..

[31] Haugen BR, Alexander EK, Bible KC, Doherty GM, Mandel SJ, Nikiforov YE, Pacini F, Randolph GW, Sawka AM, Schlumberger M, Schuff KG, Sherman SI, Sosa JA, Steward DL, Tuttle RM, Wartofsky L (2016). 2015 American Thyroid Association Management Guidelines for Adult Patients with Thyroid Nodules and Differentiated Thyroid Cancer: The American Thyroid Association Guidelines Task Force on Thyroid Nodules and Differentiated Thyroid Cancer. Thyroid.

[32] Schlumberger M, Tahara M, Wirth LJ, Robinson B, Brose MS, Elisei R, Habra MA, Newbold K, Shah MH, Hoff AO, Gianoukakis AG, Kiyota N, Taylor MH, Kim S-B, Krzyzanowska MK, Dutcus CE, De Las Heras B, Zhu J, Sherman SI (2015). Lenvatinib versus Placebo in Radioiodine-Refractory Thyroid Cancer. N. Engl. J. Med..

[33] Brose MS, Krajewska JA, Vaisman F, Hoff AO, Hitre E, Oliver J, Williamson DS, Berry N, Capdevila Castillon J (2022). 1653P Cabozantinib (C) vs placebo (P) in patients (pts) with radioiodine-refractory (RAIR) differentiated thyroid cancer (DTC) who progressed after prior VEGFR-targeted therapy: Outcomes by duration of prior lenvatinib (L) treatment. Ann. Oncol..

[34] Cabanillas ME, Brose MS, Holland J, Ferguson KC, Sherman SI (2014). A Phase I Study of Cabozantinib (XL184) in Patients with Differentiated Thyroid Cancer. Thyroid.

[35] Shah MH, De souza J, Wirth L, Menefee ME, Liu S, Geyer S, Wright J, Villalona M, Cabanillas M (2015). Cabozantinib in patients with radioiodine-refractory differentiated thyroid cancer who progressed on prior VEGFR-targeted therapy: Results of NCI-and itog-sponsored multicenter phase II clinical trial. Thyroid.

[36] M.E. Cabanillas, M.S. Brose, D.A. Ramies, Y. Lee, D. Miles, S.I. Sherman, Antitumor activity of cabozantinib (XL184) in a cohort of patients (pts) with differentiated thyroid cancer (DTC), J. Clin. Oncol. 30 (2012). https://www.embase.com/search/results?subaction=viewrecord&id=L71004222&from=export

[37] B. Konda, M.V. Knopp, P.R. Martin, S. Geyer, M.E. Cabanillas, J.A. De Souza, L.J. Wirth, M.E. Menefee, S.V. Liu, M.H. Shah, Effect of cabozantinib on bone turnover markers (BTM) and bone metastases (BM) in radioiodine refractory (RAIR)-differentiated thyroid cancer (DTC), J. Clin. Oncol. 35 (2017). https://www.embase.com/search/results?subaction=viewrecord&id=L617627373&from=export 10.1200/JCO.2017.73.0226 PMC5652872 28817373

[38] Konda B, Sherman E, Massarelli E, Xia B, Muzaffar J, Morris J, Ryder M, Ho A, Agulnik M, Wei L, Jacob R, Wright J, Streicher H, Carson W, Shah M (2022). Cabozantinib in combination with nivolumab and ipilimumab in patients with radioiodine (rai)-refractory differentiated thyroid cancer (dtc) whose cancer progressed after one prior vascular endothelial growthfactor receptor (vegfr)-targeted therapy: interim results of a multicenter phase 2 national cancer institute (nci)-international thyroid oncology group (itog) trial (nci#10240). Thyroid.

[39] Taylor M, Daniels G, Thein K, Loriot Y, Khan S, Goldschmidt J, Lebellec L, Sarantopoulos J, Vozy A, Castel-Ajgal Z, Andrianova S, Sudhagoni R, Levytskyy R, Dadu R (2022). Cabozantinib in combination with atezolizumab as a first-line therapy in patients with radioiodine-refractory (rair) differentiated thyroid cancer (dtc): results from cohort 18 of the phase 1b cosmic-21 study. Thyroid.

[40] M.S. Brose, S. Shenoy, N. Bhat, A.K. Harlacker, R.K. Yurtal, Z.A. Posey, D.M. Torrente, C. Grande, C.M. Squillante, A. Troxel, M. Yarchoan, A phase II trial of cabozantinib (CABO) for the treatment of radioiodine (RAI)-refractory differentiated thyroid carcinoma (DTC) in the first-line setting, J. Clin. Oncol. 36 (2018). 10.1200/JCO.2018.36.15-suppl.6088

[41] Xin H, Wei R, Ma Q, Wang N, Li A, Li W (2021). Merkel cell carcinoma after liver transplantation: a case report and review of the literature. Ann. Palliat. Med..

[42] Becker JC, Stang A, DeCaprio JA, Cerroni L, Lebbé C, Veness M, Nghiem P (2017). Merkel cell carcinoma. Nat. Rev. Dis. Prim..

[43] Xue Y, Thakuria M (2019). Merkel Cell Carcinoma Review. Hematol. Oncol. Clin. North Am..

[44] Iyer JG, Blom A, Doumani R, Lewis C, Tarabadkar ES, Anderson A, Ma C, Bestick A, Parvathaneni U, Bhatia S, Nghiem P (2016). Response rates and durability of chemotherapy among 62 patients with metastatic Merkel cell carcinoma. Cancer Med..

[45] Bhatia S, Storer BE, Iyer JG, Moshiri A, Parvathaneni U, Byrd D, Sober AJ, Sondak VK, Gershenwald JE, Nghiem P (2016). Adjuvant Radiation Therapy and Chemotherapy in Merkel Cell Carcinoma: Survival Analyses of 6908 Cases From the National Cancer Data Base. J. Natl. Cancer Inst..

[46] Mortier L (2003). Radiotherapy Alone for Primary Merkel Cell Carcinoma. Arch. Dermatol..

[47] Nghiem P, Bhatia S, Lipson EJ, Sharfman WH, Kudchadkar RR, Brohl AS, Friedlander PA, Daud A, Kluger HM, Reddy SA, Boulmay BC, Riker AI, Burgess MA, Hanks BA, Olencki T, Margolin K, Lundgren LM, Soni A, Ramchurren N, Church C, Park SY, Shinohara MM, Salim B, Taube JM, Bird SR, Ibrahim N, Fling SP, Homet Moreno B, Sharon E, Cheever MA, Topalian SL (2019). Durable Tumor Regression and Overall Survival in Patients With Advanced Merkel Cell Carcinoma Receiving Pembrolizumab as First-Line Therapy. J. Clin. Oncol..

[48] Tai PTH, Yu E, Winquist E, Hammond A, Stitt L, Tonita J, Gilchrist J (2000). Chemotherapy in Neuroendocrine/Merkel Cell Carcinoma of the Skin: Case Series and Review of 204 Cases. J. Clin. Oncol..

[49] H.L. Kaufman, Updated efficacy of avelumab in patients with previously treated metastatic Merkel cell carcinoma after ≥1 year of follow-up: JAVELIN Merkel 200, a phase 2 clinical trial, **6**(1), 7 (2018). 10.1186/s40425-017-0310-x PMC5774167 29347993

[50] H. Zhang, Apatinib for molecular targeted therapy in tumor, Drug Des. Devel. Ther. (2015) 6075. 10.2147/DDDT.S97235 10.2147/DDDT.S97235 PMC4654530 26622168

[51] Brunner M, Thurnher D, Pammer J, Geleff S, Heiduschka G, Reinisch CM, Petzelbauer P, Erovic BM (2008). Expression of VEGF-A/C, VEGF-R2, PDGF-a/b, c-kit, EGFR, Her-2/Neu, Mcl-1 and Bmi-1 in Merkel cell carcinoma. Mod. Pathol..

[52] Tarabadkar ES, Thomas H, Blom A, Parvathaneni U, Olencki T, Nghiem P, Bhatia S (2018). Clinical Benefit from Tyrosine Kinase Inhibitors in Metastatic Merkel Cell Carcinoma: A Case Series of 5 Patients. Am. J. Case Rep..

[53] Rabinowits G, Lezcano C, Catalano PJ, McHugh P, Becker H, Reilly MM, Huang J, Tyagi A, Thakuria M, Bresler SC, Sholl LM, Shapiro GI, Haddad R, DeCaprio JA (2018). Cabozantinib in Patients with Advanced Merkel Cell Carcinoma. Oncologist.

[54] Roman-Gonzalez A, Zhou S, Ayala-Ramirez M, Shen C, Waguespack SG, Habra MA, Karam JA, Perrier N, Wood CG, Jimenez C (2018). Impact of Surgical Resection of the Primary Tumor on Overall Survival in Patients With Metastatic Pheochromocytoma or Sympathetic Paraganglioma. Ann. Surg..

[55] Capatina C, Ntali G, Karavitaki N, Grossman AB (2013). The management of head-and-neck paragangliomas. Endocr. Relat. Cancer.

[56] Pryma DA, Chin BB, Noto RB, Dillon JS, Perkins S, Solnes L, Kostakoglu L, Serafini AN, Pampaloni MH, Jensen J, Armor T, Lin T, White T, Stambler N, Apfel S, DiPippo VA, Mahmood S, Wong V, Jimenez C (2019). Efficacy and Safety of High-Specific-Activity ^131^ I-MIBG Therapy in Patients with Advanced Pheochromocytoma or Paraganglioma. J. Nucl. Med..

[57] Niemeijer ND, Alblas G, van Hulsteijn LT, Dekkers OM, Corssmit EPM (2014). Chemotherapy with cyclophosphamide, vincristine and dacarbazine for malignant paraganglioma and pheochromocytoma: systematic review and meta-analysis. Clin. Endocrinol. (Oxf.)..

[58] van Hulsteijn LT, Niemeijer ND, Dekkers OM, Corssmit EPM (2014). ^131^ I-MIBG therapy for malignant paraganglioma and phaeochromocytoma: systematic review and meta-analysis. Clin. Endocrinol. (Oxf.)..

[59] Jimenez C (2018). Treatment for Patients With Malignant Pheochromocytomas and Paragangliomas: A Perspective From the Hallmarks of Cancer. Front. Endocrinol..

[60] O’Kane GM, Ezzat S, Joshua AM, Bourdeau I, Leibowitz-Amit R, Olney HJ, Krzyzanowska M, Reuther D, Chin S, Wang L, Brooks K, Hansen AR, Asa SL, Knox JJ (2019). A phase 2 trial of sunitinib in patients with progressive paraganglioma or pheochromocytoma: the SNIPP trial. Br. J. Cancer.

[61] Economides MP, Shah AY, Jimenez C, Habra MA, Desai M, Campbell MT (2020). A Durable Response With the Combination of Nivolumab and Cabozantinib in a Patient With Metastatic Paraganglioma: A Case Report and Review of the Current Literature. Front. Endocrinol..

[62] Apolo AB, Nadal R, Girardi DM, Niglio SA, Ley L, Cordes LM, Steinberg SM, Sierra Ortiz O, Cadena J, Diaz C, Mallek M, Davarpanah NN, Costello R, Trepel JB, Lee M-J, Merino MJ, Bagheri MH, Monk P, Figg WD, Gulley JL, Agarwal PK, Valera V, Chalfin HJ, Jones J, Streicher H, Wright JJ, Ning YM, Parnes HL, Dahut WL, Bottaro DP, Lara PN, Saraiya B, Pal SK, Stein MN, Mortazavi A (2020). Phase I Study of Cabozantinib and Nivolumab Alone or With Ipilimumab for Advanced or Metastatic Urothelial Carcinoma and Other Genitourinary Tumors. J. Clin. Oncol..

[63] Roman-Gonzalez A, Jimenez C (2017). Malignant pheochromocytoma–paraganglioma: pathogenesis, TNM staging, and current clinical trials. Curr. Opin. Endocrinol. Diabetes Obes..

[64] Fassnacht M, Dekkers OM, Else T, Baudin E, Berruti A, de Krijger RR, Haak HR, Mihai R, Assie G, Terzolo M (2018). European Society of Endocrinology Clinical Practice Guidelines on the management of adrenocortical carcinoma in adults, in collaboration with the European Network for the Study of Adrenal Tumors. Eur. J. Endocrinol..

[65] Else T, Kim AC, Sabolch A, Raymond VM, Kandathil A, Caoili EM, Jolly S, Miller BS, Giordano TJ, Hammer GD (2014). Adrenocortical Carcinoma. Endocr. Rev..

[66] T. Massimo, A. Alberto, F. Martin, D. Fulvia, T. Libuse, C.P. Antonio, R. Ruth, B. Lisa, S. Paola, G. Erika, R. Giuseppe, B. Enrico, P. Mauro, S. Wolfgang, H. Stefanie, K. Ann-Cathrin, A. Emanuela, A. Bruno, L. Paola, L. Gaetano, M. Massimo, B. Paolo, M. Franco, A. Bruno, D. Luigi, B. Alfredo, Adjuvant Mitotane Treatment for Adrenocortical Carcinoma, N. Engl. J. Med. **356**:2372–2380 (2007) 10.1056/NEJMoa063360 17554118

[67] Fassnacht M, Terzolo M, Allolio B, Baudin E, Haak H, Berruti A, Welin S, Schade-Brittinger C, Lacroix A, Jarzab B, Sorbye H, Torpy DJ, Stepan V, Schteingart DE, Arlt W, Kroiss M, Leboulleux S, Sperone P, Sundin A, Hermsen I, Hahner S, Willenberg HS, Tabarin A, Quinkler M, de la Fouchardière C, Schlumberger M, Mantero F, Weismann D, Beuschlein F, Gelderblom H, Wilmink H, Sender M, Edgerly M, Kenn W, Fojo T, Müller H-H, Skogseid B (2012). Combination Chemotherapy in Advanced Adrenocortical Carcinoma. N. Engl. J. Med..

[68] Berruti A, Sperone P, Ferrero A, Germano A, Ardito A, Priola AM, De Francia S, Volante M, Daffara F, Generali D, Leboulleux S, Perotti P, Baudin E, Papotti M, Terzolo M (2012). Phase II study of weekly paclitaxel and sorafenib as second/third-line therapy in patients with adrenocortical carcinoma. Eur. J. Endocrinol..

[69] Phan LM, Fuentes-Mattei E, Wu W, Velazquez-Torres G, Sircar K, Wood CG, Hai T, Jimenez C, Cote GJ, Ozsari L, Hofmann M-C, Zheng S, Verhaak R, Pagliaro L, Cortez MA, Lee M-H, Yeung S-CJ, Habra MA (2015). Hepatocyte Growth Factor/cMET Pathway Activation Enhances Cancer Hallmarks in Adrenocortical Carcinoma. Cancer Res..

[70] Kroiss M, Megerle F, Kurlbaum M, Zimmermann S, Wendler J, Jimenez C, Lapa C, Quinkler M, Scherf-Clavel O, Habra MA, Fassnacht M (2020). Objective Response and Prolonged Disease Control of Advanced Adrenocortical Carcinoma with Cabozantinib. J. Clin. Endocrinol. Metab..

[71] Miller KC, Chintakuntlawar AV, Hilger C, Bancos I, Morris JC, Ryder M, Smith CY, Jenkins SM, Bible KC (2020). Salvage Therapy With Multikinase Inhibitors and Immunotherapy in Advanced Adrenal Cortical Carcinoma. J. Endocr. Soc..

[72] Grillo F, Florio T, Ferraù F, Kara E, Fanciulli G, Faggiano A, Colao A (2018). Emerging multitarget tyrosine kinase inhibitors in the treatment of neuroendocrine neoplasms. Endocr. Relat. Cancer.

[73] Garcia-Carbonero R, Sorbye H, Baudin E, Raymond E, Wiedenmann B, Niederle B, Sedlackova E, Toumpanakis C, Anlauf M, Cwikla JB, Caplin M, O”Toole D, Perren A, all other Vienna Consensus Conference participants (2016). ENETS Consensus Guidelines for High-Grade Gastroenteropancreatic Neuroendocrine Tumors and Neuroendocrine Carcinomas. Neuroendocrinology.

[74] Pavel M, O’Toole D, Costa F, Capdevila J, Gross D, Kianmanesh R, Krenning E, Knigge U, Salazar R, Pape U-F, Öberg K, all other Vienna Consensus Conference participants (2016). ENETS Consensus Guidelines Update for the Management of Distant Metastatic Disease of Intestinal, Pancreatic, Bronchial Neuroendocrine Neoplasms (NEN) and NEN of Unknown Primary Site. Neuroendocrinology.

[75] Yao JC, Hassan M, Phan A, Dagohoy C, Leary C, Mares JE, Abdalla EK, Fleming JB, Vauthey J-N, Rashid A, Evans DB (2008). One Hundred Years After “Carcinoid”: Epidemiology of and Prognostic Factors for Neuroendocrine Tumors in 35,825 Cases in the United States. J. Clin. Oncol..

[76] Raymond E, Dahan L, Raoul J-L, Bang Y-J, Borbath I, Lombard-Bohas C, Valle J, Metrakos P, Smith D, Vinik A, Chen J-S, Hörsch D, Hammel P, Wiedenmann B, Van Cutsem E, Patyna S, Lu DR, Blanckmeister C, Chao R, Ruszniewski P (2011). Sunitinib Malate for the Treatment of Pancreatic Neuroendocrine Tumors. N. Engl. J. Med..

[77] Reuther C, Heinzle V, Spampatti M, Vlotides G, De Toni E, Spöttl G, Maurer J, Nölting S, Göke B, Auernhammer CJ (2016). Cabozantinib and Tivantinib, but Not INC280, Induce Antiproliferative and Antimigratory Effects in Human Neuroendocrine Tumor Cells in vitro: Evidence for “Off-Target” Effects Not Mediated by c-Met Inhibition. Neuroendocrinology.

[78] Sennino B, Ishiguro-Oonuma T, Wei Y, Naylor RM, Williamson CW, Bhagwandin V, Tabruyn SP, You W-K, Chapman HA, Christensen JG, Aftab DT, McDonald DM (2012). Suppression of Tumor Invasion and Metastasis by Concurrent Inhibition of c-Met and VEGF Signaling in Pancreatic Neuroendocrine Tumors. Cancer Discov..

[79] J.A. Chan, J.E. Faris, J.E. Murphy, L.S. Blaszkowsky, E.L. Kwak, N.J. McCleary, C.S. Fuchs, J.A. Meyerhardt, K. Ng, A.X. Zhu, T.A. Abrams, B.M. Wolpin, S. Zhang, A. Reardon, B. Fitzpatrick, M.H. Kulke, D.P. Ryan, Phase II trial of cabozantinib in patients with carcinoid and pancreatic neuroendocrine tumors (pNET), J. Clin. Oncol. 35 (2017). https://www.embase.com/search/results?subaction=viewrecord&id=L618086990&from=export

[80] Das S, Dasari A (2021). Novel therapeutics for patients with well-differentiated gastroenteropancreatic neuroendocrine tumors. Ther. Adv. Med. Oncol..

[81] Feliu J, Heredia-Soto V, Gironés R, Jiménez-Munarriz B, Saldaña J, Guillén-Ponce C, Molina-Garrido MJ (2020). Management of the toxicity of chemotherapy and targeted therapies in elderly cancer patients. Clin. Transl. Oncol..

[82] Mohile SG, Hardt M, Tew W, Owusu C, Klepin H, Gross C, Gajra A, Lichtman SM, Feng T, Togawa K, Ramani R, Katheria V, Hansen K, Hurria A, Cancer and Aging Research Group. (2013). Toxicity of Bevacizumab in Combination with Chemotherapy in Older Patients. Oncologist.

[83] Smith M, De Bono J, Sternberg C, Le Moulec S, Oudard S, De Giorgi U, Krainer M, Bergman A, Hoelzer W, De Wit R, Bögemann M, Saad F, Cruciani G, Thiery-Vuillemin A, Feyerabend S, Miller K, Houédé N, Hussain S, Lam E, Polikoff J, Stenzl A, Mainwaring P, Ramies D, Hessel C, Weitzman A, Fizazi K (2016). Phase III Study of Cabozantinib in Previously Treated Metastatic Castration-Resistant Prostate Cancer: COMET-1. J. Clin. Oncol..

