# Supplementary material for: Cabozantinib for different endocrine tumours: killing two birds with one stone. A systematic review of the literature
Source: Endocrine. 2023 Oct 18;83(1):26–40. doi: 10.1007/s12020-023-03526-0 (PMC10805963; doi:10.1007/s12020-023-03526-0)
Supplement: Supplementary file 1 [file 12020_2023_3526_MOESM1_ESM.doc]

# Online Supplemental File 1 (S1)

**Cabozantinib for different endocrine tumours: killing two birds with one stone. A systematic review of the literature**

**.**

**(Protocol and Appendix)**

Elena Zago^1^, Antonio Galluzzo^2^, Silvia Pradella^2^, Lorenzo Antonuzzo^3^, Mario Maggi^1^

Luisa Petrone^1^, Clotilde Sparano^1^

^1^ Endocrinology Unit, Department of Experimental and Clinical Biomedical Sciences 'Mario Serio', University of Florence, Florence, Italy

^2^ Department of Radiology, Careggi University Hospital, Florence, Italy

^3^ Department of Oncology, Careggi University Hospital, Florence, Italy

Corresponding author: Dr Luisa Petrone, MD, PhD

Careggi University Hospital

Florence, Italy

luisa.petrone@unifi.it

1. **Background**

Merkel cell carcinoma (MCC) is a rare, highly aggressive neuroendocrine skin tumour that usually develops in sun-exposed areas (1). It affects elderly subjects with a story of sun exposition and/or immunosuppressant therapy (2),(3). Local and regional lymph node metastases can occur at the early stages, and the long-term prognosis is poor, with a five-year survival which drops to 14% in the event of metastatic disease (4). For locoregional disease, first-line therapy encompasses tumour excision, sentinel lymph node dissection and/or lymphadenectomy, followed by high-dose adjuvant radiotherapy to the primary site. Since cytotoxic chemotherapy failed to show prolonged tumour response, along with the occurrence of severe adverse events, immune-checkpoint inhibitors (PD-1 and PDL-1 ligand inhibitors) became the favoured agents for advanced disease (5),(6) (7). Despite the success of the immunotherapy, a subset of patients failed to respond. Hence, tyrosine kinase inhibitors (TKI) have been proposed as a potential treatment, due to their ability to inhibit tumour angiogenesis and spreading processes (8)(9). Cabozantinib is a small molecular inhibitor of several tyrosine kinases such as vascular endothelial growth factor receptor (VEGFR) 2, AXL, mesenchymal-epidermal transition (MET) factor, and rearranged during transfection (REF), targeting key pathways necessary to tumour survival, angiogenesis, epithelial to mesenchymal transition and other metastatic processes (10). In particular, while VEGFR inhibition alone can lead to a tumour invasion because of the hypoxia-induced MET pathway activation, cabozantinib can block both pathways concurrently resulting in enhanced efficacy in disease control (10).

1. **Objectives**

The present systematic review aims to identify and summarize the role of cabozantinib therapy in endocrine and neuroendocrine tumours. Using the PICO process (11), we have phrased the following research questions:

- 1. **Primary Objectives**

1. In patients with endocrine tumours (e.g. medullary thyroid cancer, radioiodine refractory differentiated thyroid cancer, pheochromocytoma, paraganglioma, adrenocortical carcinoma, etc.) (P), did cabozantinib (I), compared to other therapy (C) show any efficacy in terms of progression-free survival or objective response (O)?
2. In particular, does cabozantinib (P) have a role (I), besides radio and/or chemotherapy and/or inhibitor of immune checkpoint (C), in improving the progression-free survival of patients suffering from MCC (O)?
   1. **Secondary Objectives**
3. Are there any trials (P) actively recruiting patients suffering from endocrine and neuroendocrine tumours (I) that are comparing cabozantinib vs. other treatment/placebo (C) assessing progression-free survival (O)?
4. **Methods**
   1. **Systematic literature review**

For each PICO question a systematic review of the literature using a predefined search strategy will be performed. Pertinent studies will be identified, their eligibility assessed and data relevant to the PICO question extracted.

**Types of studies**

We will evaluate all interventional trials, including in vivo and in vitro experiments; cross-sectional or longitudinal, retrospective or prospective, observational clinical and research studies as well as case reports, case series, expert opinions, meta-analyses and review of the current literature. We will include only studies published in English and listed in PubMed, Scopus and EMBASE since 2009.

*Participants*

*Inclusion criteria*

1. Patients aged ≥18 years with endocrine and neuroendocrine tumours, including differentiated thyroid carcinoma, medullary thyroid carcinoma, adrenocortical carcinoma, pheochromocytoma, paraganglioma, gastroenteropancreatic and lung neuroendocrine tumours as well as MCC.
2. In vivo and in vitro experiments on endocrine and neuroendocrine tumour models treated with cabozantinib

*Exclusion criteria*

1. Paediatric population
2. Patients suffering from neuroendocrine tumours arising from the genitourinary tract (e.g. prostate cancers, bladder cancer etc.)

**Interventions**

We will include studies with patients, animal models and cell models treated with cabozantinib alone and cabozantinib plus other therapies for endocrine and neuroendocrine tumours. In addition, we will focus on the use of cabozantinib in MCC.

**Electronic literature search**

We will search MEDLINE (PubMed), EMBASE and Scopus for relevant English literature from 2009 to 2023. The search will be updated shortly before submission of the planned manuscript in order to also include the newest references. The search strategy will be developed in accordance with the clinical question.

We will use the following search terms: “cabozantinib”, AND/OR “adrenocortical carcinoma”, “pheochromocytoma”, “paraganglioma”, “Merkel cell carcinoma”, “neuroendocrine tumour*”, “carcinoid*”, “neuroendocrine neoplasia”, “neuroendocrine neoplasm*”.

Non-English literature will not be included. Reports exclusively dealing with data on paediatric patients (age <18 years) will not be included. The references of relevant articles will be manually searched to identify additional articles. Further, papers will be cross-referenced using the ‘cited by’ function on PubMed. If necessary, personal communication with authors will be attempted via email or phone in order to obtain additional relevant data.

- 1. **Data collection and analysis**

**Selection of studies**

Titles will be reviewed at first, followed by an evaluation of the abstracts with titles suggesting that a study might be of relevance. Eligible studies will be identified based on their full text. All potentially relevant articles will be reviewed and confirmed for inclusion. We will use proprietary reference manager software to manage a large number of studies, and we will document the study selection in a detailed flow chart.

**Data extraction and management**

For the studies we will record 1) investigated condition 2) principal investigator 3) publication date 4) study design (e.g. experimental or clinical; cross-sectional or longitudinally), 5) trial phase (if applicable) 6) number of enrolled patients, 7) dose of cabozantinib.

Data will be extracted by the first author and cross-checked by the senior authors; any uncertainties concerning data extraction and interpretation will be resolved by consensus by the senior authors.

For the active trials we will record 1) the investigated condition, 2) the identifier number, 3) recruitment status, 4) the study starts and completion date, 5) the study phase, 6) the number of enrolled patients, 7) the primary objectives, 8) investigated drugs.

*Investigations of heterogeneity*

We will attempt to explore possible sources of heterogeneity. These will likely be related to variances in methods of clinical diagnosis, inclusion criteria and heterogeneity in cabozantinib dosage, titration and use as a second- or third-line therapy.

- 1. **Publication and authorship**

Authorship will be defined according to the Vancouver criteria.

- 1. **Funding**

None

### References

1. Xin H, Wei R, Ma Q, Wang N, Li A, Li W. Merkel cell carcinoma after liver transplantation: a case report and review of the literature. Ann Palliat Med. 2021 Jul;10(7):8425–30.

2. Xue Y, Thakuria M. Merkel Cell Carcinoma Review. Hematology/Oncology Clinics of North America. 2019 Feb;33(1):39–52.

3. Iyer JG, Blom A, Doumani R, Lewis C, Tarabadkar ES, Anderson A, et al. Response rates and durability of chemotherapy among 62 patients with metastatic Merkel cell carcinoma. Cancer Med. 2016 Sep;5(9):2294–301.

4. Coggshall K, Tello TL, North JP, Yu SS. Merkel cell carcinoma: An update and review. Journal of the American Academy of Dermatology. 2018 Mar;78(3):433–42.

5. Bhatia S, Storer BE, Iyer JG, Moshiri A, Parvathaneni U, Byrd D, et al. Adjuvant Radiation Therapy and Chemotherapy in Merkel Cell Carcinoma: Survival Analyses of 6908 Cases From the National Cancer Data Base. JNCI J Natl Cancer Inst. 2016 Sep;108(9):djw042.

6. Mortier L. Radiotherapy Alone for Primary Merkel Cell Carcinoma. Arch Dermatol. 2003 Dec 1;139(12):1587.

7. Tai PTH, Yu E, Winquist E, Hammond A, Stitt L, Tonita J, et al. Chemotherapy in Neuroendocrine/Merkel Cell Carcinoma of the Skin: Case Series and Review of 204 Cases. JCO. 2000 Jun 12;18(12):2493–9.

8. Nghiem P, Bhatia S, Lipson EJ, Sharfman WH, Kudchadkar RR, Brohl AS, et al. Durable Tumor Regression and Overall Survival in Patients With Advanced Merkel Cell Carcinoma Receiving Pembrolizumab as First-Line Therapy. JCO. 2019 Mar 20;37(9):693–702.

9. Zhang H. Apatinib for molecular targeted therapy in tumor. DDDT. 2015 Nov;6075.

10. Yakes FM, Chen J, Tan J, Yamaguchi K, Shi Y, Yu P, et al. Cabozantinib (XL184), a Novel MET and VEGFR2 Inhibitor, Simultaneously Suppresses Metastasis, Angiogenesis, and Tumor Growth. Molecular Cancer Therapeutics. 2011 Dec 1;10(12):2298–308.

11. Schardt C, Adams MB, Owens T, Keitz S, Fontelo P. Utilization of the PICO framework to improve searching PubMed for clinical questions. BMC Med Inform Decis Mak. 2007 Dec;7(1):16.

###

**Appendix**

**Full electronic search strategy for Pubmed:**

((((((((((("differentiated thyroid cancer") OR ("papillary thyroid cancer")) OR ("medullary thyroid cancer")) OR (pheochromocytoma)) OR (paraganglioma)) OR ("adrenocortical carcinoma")) OR ("merkel cell carcinoma")) OR ("neuroendocrine tumor")) OR ("neuroendocrine neoplasm")) OR ("neuroendocrine neoplasms")) OR ("neuroendocrine tumors")) AND (cabozantinib)
